# Flexible spectral imaging color enhancement in colon capsule endoscopy: Scoping review of evidence for lesion detection and characterization

**DOI:** 10.1055/a-2821-8380

**Published:** 2026-03-16

**Authors:** Pablo Cortegoso Valdivia, Ervin Toth, Anastasios Koulaouzidis

**Affiliations:** 1 18630 Gastroenterology and Endoscopy Unit, University Hospital of Parma, Parma, Italy; 2 11286 Surgical Research Unit, Odense University Hospital, Svendborg, Denmark; 3 6174 Department of Clinical Research, University of Southern Denmark, Odense, Denmark; 4 Department of Gastroenterology, Skåne University Hospital, Lund University, Malmö, Sweden; 5 11286 Department of Medicine, Odense University Hospital, Svendborg, Denmark; 6 37805 Department of Social Medicine & Public Health, Pomeranian Medical University in Szczecin, Szczecin, Poland

**Keywords:** Endoscopy Lower GI Tract, Diagnosis and imaging (inc chromoendoscopy, NBI, iSCAN, FICE, CLE...), Endoscopy Small Bowel, Capsule endoscopy, GI Pathology, Polyps / adenomas / ...

## Abstract

**Background and study aims:**

Second-generation colon capsule endoscopy (CCE) has achieved high diagnostic accuracy for polyp detection. However, its widespread adoption as a filter test is hampered by major cost-effectiveness concerns, due to significant follow-up endoscopy rates (FER), quantified as high as 42%, thus revealing a potential paradox: CCE superior detection of un-characterized lesions may worsen the economic problem. Flexible spectral imaging color enhancement (FICE) is a standard virtual chromoendoscopy tool, but its role in CCE has been considered uncertain. This scoping review aimed to evaluate available evidence on FICE in addressing CCE limitations in lesion detection and characterization.

**Methods:**

A systematic literature search identified studies investigating use of FICE in CCE, focusing on studies analyzing FICE performance in improving detection rates and differentiating polyp histology.

**Results:**

Two key studies representing complementary evidence were analyzed. Regarding detection, a prospective trial demonstrated that FICE significantly improved overall per-lesion sensitivity compared with white light (WL) (79% vs. 61%). Specifically, FICE outperformed WL for 6-9-mm polyps (93% vs. 65%), flat/non-protruding lesions (75% vs. 53%), and serrated polyps (74% vs. 57%). Regarding characterization, a quantitative colorimetric analysis identified that the color difference between polyps and mucosa in FICE reliably discriminates histology, yielding a 0.928 area-under-the-curve (AUC), with 91.2% sensitivity and 88.2% specificity.

**Conclusions:**

FICE may play a complementary role in CCE, significantly improving detection of high-risk flat/serrated lesions and providing objective data for differentiating adenomas from hyperplastic polyps. Integrating FICE into diagnostic workflows could provide a smart solution to address CCE high FER and cost-effectiveness barriers.

## Introduction

Second-generation colon capsule endoscopy (CCE) is a reliable technology for detection of colorectal lesions 1 2 . A recent systematic meta-review by Lei et al. confirmed its high diagnostic accuracy, with per-patient sensitivity and specificity for ≥ 6-mm polyps approaching 80% and 87%, respectively, and 88% and 95% for ≥ 10 mm polyps 3 . Despite this technical success, its widespread clinical adoption remains slow 4 . Real-world data from large-scale initiatives in the UK (NHS England, ScotCap) and Denmark (CareForColon) paint a complex picture of variable protocols, unsatisfactory completion rates, and, most importantly, high rates of subsequent invasive procedures 5 .

The core barrier to CCE implementation is its cost-effectiveness. This barrier was recently quantified in a 2025 meta-analysis by Lei et al, which found the pooled follow-up endoscopy rate (FER) (i.e., the rate of patients requiring a subsequent colonoscopy or sigmoidoscopy) to be 42% 6 . This undermines CCE's primary value proposition as a scalable cost-effective filter test. The same paper revealed a “second-generation CCE paradox” because the FER for the older CCE1 was 27%, whereas the FER for the newer CCE2 was significantly higher at 48% 6 , suggesting that the technological improvement in detection paradoxically led to a worsening in cost-effectiveness because every additional (un-characterized) lesion found triggers a potential referral for an invasive, expensive procedure.

These data unveil the “bottleneck” of the CCE pathway, highlighting that the problem is not one of detection but of characterization, and exposing a quality gap. First, white-light (WL) imaging suffers from inability to predict histology, as confirmed by the Nyborg Consensus expert panel. Although readers should use polyp classification and pit-pattern analysis borrowed from colonoscopy, it is often impossible with CCE WL images due to underwater imaging, lack of magnification, and lack of advanced chromoendoscopic imaging like narrow band imaging (NBI) 7 . The panel explicitly identified “reliable polyp pathology prediction” as a top research priority 7 . Second, this lack of characterization leads to inability to match polyps. This, combined with unreliable localization, creates significant challenges in both internal matching (counting the same polyp multiple times) and external matching (confirming a CCE finding during colonoscopy), driving up false-positive rates and, consequently, the FER 8 . To break this cycle, CCE must evolve from a simple detection tool to a diagnostic tool.

**Fig. 1:**
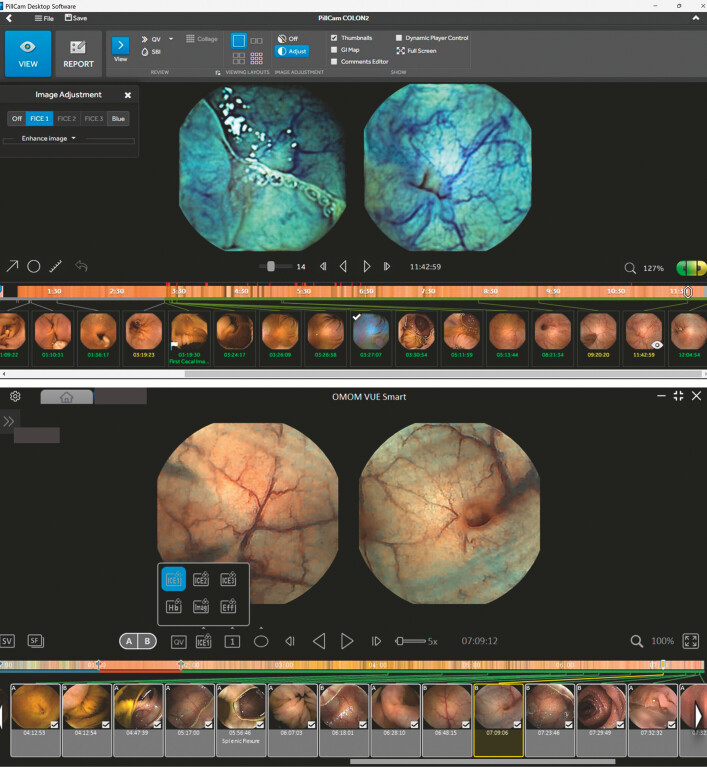
PillCam RAPID Reader software, Medtronic (upper row), and OMOM VUE Smart software, Jinshan Science & Technology (lower row), with activated image adjustment tools.

Flexible spectral imaging color enhancement (FICE) is an algorithmic, post-processing image enhancement that has been an integrated software feature on capsule platforms for over a decade. Originally developed by Fujifilm (Tokyo, Japan) and branded as Fujinon Intelligent Color Enhancement, this technology is currently incorporated into the Pillcam RAPID Reader software by Medtronic (Dublin, Ireland). Similar virtual chromoendoscopy tools have been developed by other manufacturers, such as ICE (Image Color Enhancement) by Jinshan Science & Technology (Chongqing, China), incorporated into the OMOM VUE Smart software. Although these tools share the same post-processing capability, the main differences lie in proprietary algorithms used for wavelength estimation and specific color display presets available ( Fig. 1 ).

In small-bowel CE, the role of FICE is well-defined. Multiple studies and meta-analyses have concluded that although FICE does not improve the overall detection rate for small-bowel lesions, it may serve as a useful adjunct for delineating and characterizing specific lesions, most notably angioectasias (using the FICE 1 setting) 9 . On the other hand, the role of FICE in CCE represents a research gap. A 2024 international RAND consensus on polyp matching explicitly discussed FICE: the panel did not reach an agreement on its utility, labeling its role “uncertain” and citing a “general uncertainty surrounding the effectiveness of FICE” and “conflicting evidence” from the initial pilot studies 10 . However, this perceived conflict may stem from comparing studies with distinct primary endpoints. Therefore, the aim of this scoping review was to map available evidence to investigate the complementary, rather than conflicting, role of FICE in addressing the dual challenge of lesion detection and characterization.

## Search strategy and selection criteria

To identify relevant studies for this scoping review, we adopted the PRISMA-ScR (Preferred Reporting Items for Systematic reviews and Meta-Analyses extension for Scoping Reviews) guidelines 11 . We conducted a systematic search on PubMed up to September 2025 using the search terms “colon capsule endoscopy” combined with “FICE,” “Flexible Spectral Imaging Color Enhancement,” or “chromoendoscopy”. Only studies specifically analyzing the performance of FICE in CCE were included. Specific study types (case reports, reviews, letters, editorials) and studies analyzing chromoendoscopy techniques other than FICE were excluded. The initial search yielded 92 results. After screening titles and abstracts, five studies were assessed for eligibility: three were excluded (focus on small bowel only or use of non-FICE chromoendoscopy), leaving two studies included in the qualitative synthesis 12 13 .

### FICE for lesion detection

Omori et al. 12 conducted a prospective, single-center trial (89 patients) to explore whether CCE diagnostic yield with FICE was higher than with WL. According to the results, FICE significantly increased overall per-lesion sensitivity from 61% (WL) to 79% (FICE) ( *P* < 0.0001), although no significant advantage for detecting cancers (100% vs 100%) or polyps ≥ 10 mm (94% FICE vs 81% WL, *P* = 0.11) was seen in the subgroup analysis.

FICE showed significant performance in the diagnosis of small and flat colorectal lesions, which are known to be the most challenging category to identify in CCE. In detail, FICE per-lesion sensitivity outperformed WL for 6- to 9-mm polyps (93% vs. 65%, *P* = 0.0007), non-protruding/flat lesions (75% vs. 53%, *P* < 0.0001), and serrated polyps (74% vs. 57%, *P* = 0.0022).

These results are clinically relevant because such lesions (especially flat, serrated polyps) represent a significant portion of interval colorectal cancer precursors, due to their subtle morphologic features which pose inherent diagnostic constraints even in high-definition colonoscopy. Accentuation of vascular patterns and color contrast by FICE in underwater CCE images is likely to play a role in improved detection of these lesions. On the other hand, an increase in sensitivity comes with a reduction in specificity, highlighted by a high number of false positives, especially in case of suboptimal bowel preparation. Nevertheless, the net shift in favor of increased detection of subtle, clinically meaningful lesions suggests a primary role for FICE in reducing CCE’s false negative examinations.

### FICE for lesion characterization

The study by Nakazawa et al 13 addressed the difficulty of WL imaging in reliably differentiating adenomas from hyperplastic polyps. Rather than relying on subjective human interpretation, the authors applied an objective, quantitative colorimetric approach using CIELAB color space. The results demonstrated that FICE selectively amplifies color contrast of adenomatous polyps, whereas hyperplastic polyps show little to no enhancement. This phenomenon, quantified using the ΔE’ ratio (measuring the color difference between a polyp and the surrounding mucosa in both WL and FICE), proved to be an excellent discriminator for histology, yielding a 0.928 area under the curve (AUC), 91.2% sensitivity and 88.2% specificity.

These results demonstrate that FICE-processed images contain objective, measurable data required to predict histology with significant accuracy. In addition, the derived FICE ΔE’ cutoff of 1.758 offers an objective threshold potentially operationalized within reading software or incorporated into machine-learning algorithms. The clinical implication in this setting is the possibility of drastically reducing the number of subsequent unnecessary colonoscopies for small, distal polyps with no proven oncologic potential, with a direct implication on the FER.

## Implications for clinical practice and implementation pathways

**Fig. 2:**
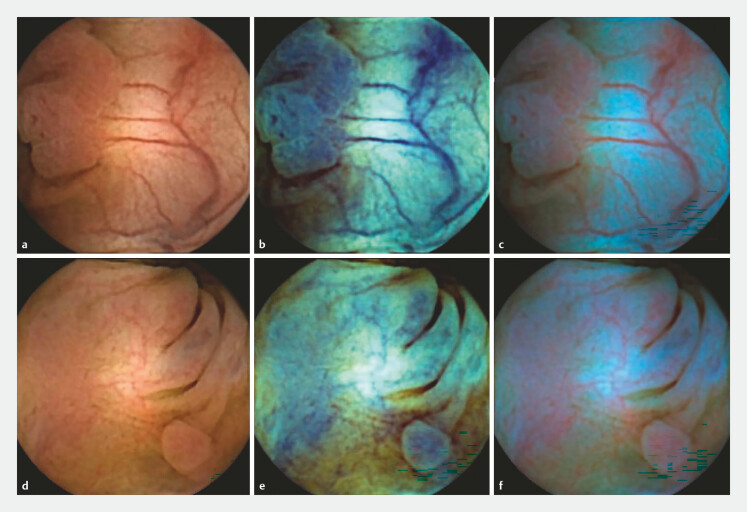
Colon capsule endoscopic (PillCam COLON2, Medtronic) images showing adenomatous (upper row) and hyperplastic (lower row) polyps. **a** , **d** Standard white-light imaging. **b** , **e** Corresponding views using FICE. **c** , **f** The same lesions visualized with Blue mode.

The dual advantage highlighted by the Japanese studies (i.e., improvement in quantity of detected clinically relevant lesions and quality of lesion characterization) directly targets the central bottleneck in current CCE workflows. High sensitivity alone, without reliable characterization, paradoxically increases FER because every detected lesion, regardless of clinical relevance, triggers referral: Improved detection is meaningful only if paired with the ability to distinguish lesions that require mandatory subsequent examinations ( Fig. 2 ).

The possibility of integrating quantitative colorimetric analysis into CCE reading software brings impactful consequences: Automated segmentation, objective ΔE’ calculation, and artificial intelligence (AI)-based classification could provide a standardized, reproducible characterization layer. Indeed, the relatively simple pixel-based color analysis used by Nakazawa et al. lends itself well to automation. This transformation could steer the role of CCE from a detection-based filter test toward a diagnostic tool capable of informing management decisions, thus revising the 1-to-1 referral cycle and answering the clinical call for characterization 7 and the economic call for cost-effectiveness 6 8 .

All these aspects imply several practical considerations, regarding workflow integration and quality standards. A logical approach would involve primary reading under FICE, capitalizing on its higher lesion detection, followed by (semi)automated ΔE’-based characterization. On the other hand, reading protocols may need revision to ensure that readers appropriately balance sensitivity with artifact recognition. In addition, the visual difference in FICE mode may be subtle to the human eye due to CCE image resolution limits (compared with optical colonoscopy). This reinforces the importance of the quantitative approach proposed by Nakazawa et al., overcoming limitations of subjective visual interpretation of lower-quality images with an objective computer-aided distinction.

Although promising, the two Japanese studies have specific limitations. The Omori study, although showing superior sensitivity of FICE, also hinted at lower specificity and high variability in interobserver agreement for intermediate-sized lesions, indicating that human interpretation remains challenging, even with enhanced imaging 12 . On the other hand, the Nakazawa study was a small, retrospective analysis. In addition, rare serrated subtypes (e.g., sessile serrated adenomas and traditional serrated adenoma) were largely excluded, raising important questions about generalizability of ΔE’ to the full spectrum of serrated neoplasia 13 . Nevertheless, these limitations do not limit alignment of both studies in demonstrating the potential of FICE in transforming the visual and quantitative landscape of CCE imaging ( Table 1 ).

**Table:** **Table 1** Summary of key studies investigating FICE in colon capsule endoscopy.

Author (year)	Design	N	Aim	Key findings	Limitations
Omori et al. (2024)	Prospective, single-center	89	Detection (sensitivity)	FICE significantly improved overall per-lesion sensitivity vs WL (79% vs 61%). Superior detection for 6-to 9-mm, flat, and serrated lesions.	Single-center design. Lower specificity with FICE compared with WL. High interobserver variability.
Nakazawa et al. (2021)	Retrospective, quantitative analysis	34	Characterization (differentiation)	Quantitative colorimetric analysis distinguished adenomas from hyperplastic polyps with 91.2% sensitivity.	Small sample size. Retrospective design. Analysis focused on differentiating adenomas vs. hyperplastic polyps (SSLs not specifically addressed).
FICE, Flexible spectral imaging color enhancement; SSL, sessile serrated lesion; WL, white light.					

## Conclusions

Evidence on the use of FICE in CCE, although possibly presenting a compelling solution to the “quality gap” of WL, currently remains limited by single-center designs and small populations. However, it may represent a crucial evolution in maturation of CCE technology, bridging the longstanding quality gap between capsule imaging and optical colonoscopy. A pragmatic approach for future research would be to conduct nested studies within the existing large-scale national pilots. Retrospective application of FICE to extensive datasets would allow for validation of the technology in this setting without need for additional patient recruitment. As capsule platforms and AI-driven tools continue to advance, integration of FICE in the diagnostic workflows may facilitate a model in which CCE not only identifies lesions but also stratifies them, with the aim of reducing unnecessary follow-up procedures, improving cost-effectiveness, and bringing capsule-based colorectal imaging closer to a complete diagnostic modality.
